# National Norms for the Elixhauser and Charlson Comorbidity Indexes Among Hospitalized Adults

**DOI:** 10.1093/geroni/igab046.2282

**Published:** 2021-12-17

**Authors:** Christine Loyd, Garner Boogaerts, Yue Zhang, Richard Kennedy, Cynthia Brown

**Affiliations:** 1 University of Alabama at Birmingham, Birmingham, Alabama, United States; 2 University of Alabama at Birmingham, University of Alabama at Birmingham, Alabama, United States; 3 Louisiana State University Health Sciences Center - New Orleans, New Orleans, Louisiana, United States

## Abstract

Multimorbidity has become the defining focus of in-patient geriatric clinical practice and research. Comorbidity assessment burden is often completed using the Elixhauser (ECI) and Charlson comorbidity indexes (CCI), which can predict mortality risk, hospital length of stay and readmission, and healthcare utilization. Yet, the national norms for ECI and CCI have not been reported. Therefore, this study aimed to report comorbidity score national norms of hospitalized patients based on age, race, and sex. Using the 2017 US National Inpatient Sample, ICD-10 coding data from 7,159,694 adult patient’s (≥18years) was abstracted to calculate ECI and CCI scores. Scores were stratified into 5-year age increments from age 45-89. Adults aged<45 and >89 were included in the analysis, however not age-stratified. Overall mean comorbidity score for the population using the ECI was 2.76 (95%CI 2.76, 2.76) and CCI was 1.22 (95% CI 1.22, 1.22). Mean scores for both indexes increased with age until age 90, and this increase was independent of race and sex (all p-values<0.001). Some individual comorbidities increased with age including congestive heart failure and dementia, while others including diabetes and chronic obstructive pulmonary disease increased with age but peaked between 60-74 years and declined in older age. Importantly, a report of US national norms for comorbidity burden among hospitalized adults can provide a reference for determining if clinical and research populations have greater or lesser comorbidity than typical hospitalized adults for their age, race, and sex.

